# The cancer-testis antigens SPANX-A/C/D and CTAG2 promote breast cancer invasion

**DOI:** 10.18632/oncotarget.7408

**Published:** 2016-02-15

**Authors:** Erin A. Maine, Jill M. Westcott, Amanda M. Prechtl, Tuyen T. Dang, Angelique W. Whitehurst, Gray W. Pearson

**Affiliations:** ^1^ Harold C. Simmons Cancer Center, University of Texas Southwestern Medical Center, Dallas, Texas, USA; ^2^ The Department of Pharmacology, University of Texas Southwestern Medical Center, Dallas, Texas, USA

**Keywords:** cancer-testis antigen, invasion, metastasis, breast cancer, extracellular matrix

## Abstract

Genes that are normally biased towards expression in the testis are often induced in tumor cells. These gametogenic genes, known as cancer-testis antigens (CTAs), have been extenstively investigated as targets for immunotherapy. However, despite their frequent detection, the degree to which CTAs support neoplastic invasion is poorly understood. Here, we find that the CTA genes SPANX-A/C/D and CTAG2 are coordinately induced in breast cancer cells and regulate distinct features of invasive behavior. Our functional analysis revealed that CTAG2 interacts with Pericentrin at the centrosome and is necessary for directional migration. Conversely, SPANX-A/C/D interacts with Lamin A/C at the inner nuclear membrane and is required for the formation of actin-rich cellular protrusions that reorganize the extracellular matrix. Importantly, SPANX-A/C/D was required for breast cancer cells to spontaneously metastasize to the lung, demonstrating that CTA reactivation can be critical for invasion dependent phenotypes in vivo. Moreover, elevated SPANX-A/C/D expression in breast cancer patient tumors correlated with poor outcome. Together, our results suggest that distinct CTAs promote tumor progression by regulating complementary cellular functions that are integrated together to induce invasive behavior.

## INTRODUCTION

The local invasion of tumor cells into connective tissue is a key event during tumor progression that can lead to metastasis and poor patient outcome [1]. Because epithelial tissue is composed of static, adherent and polarized cells, the induction of carcinoma invasion frequently involves a change in tumor cell state. For instance, the activation of epithelial-to-mesenchymal transition (EMT) programs can induce invasion by suppressing cell-cell adhesion genes [2–4]. In addition, elevated expression of podoplanin in epithelial-like pancreatic tumor cells promotes remodeling of the actin cytoskeleton and collective invasion [5]. Moreover, rapidly migrating tumor cells near the tumor vasculature express higher levels of core cytoskeletal regulatory genes and cell surface receptors that detect chemotactic signals [6]. Given the critical role that alterations in gene expression have in promoting invasive phenotypes, we sought to further define the nature of anomalously expressed genes that promote invasive behavior.

We recently discovered an epigenetically distinct subpopulation of breast cancer “trailblazer” cells that has an enhanced ability to invade in organotypic culture and spontaneously metastasize to the lungs [7]. To prioritize genes for investigation as potential regulators of invasion, we used significance analysis of microarrays (SAM) to identify genes that were more highly expressed in the SUM159 trailblazer subpopulation compared to their relatively less invasive sibling SUM159 non-trailblazer cells. With this approach, we identified 239 probesets corresponding to 205 genes that were more highly expressed in the SUM159 trailblazer cells. Analysis of the attributes of these genes revealed that 28 probesets detected gametogenic genes that have been classified as cancer/testis antigens (CTAs). Genes categorized as CTAs are normally biased towards expression in the testis and are not expressed in adult female tissue [8]. However, CTAs are frequently induced in response to epigenetic aberrations in various cancer types, including breast, lung, ovarian, bladder and melanoma tumors [9]. Thus, the CTAs detected in the invasive trailblazer cells were a set of aberrantly expressed genes that had the potential to regulate invasive traits.

CTAs share a biased expression profile; however, the coding sequences of the known CTAs show significant variability, which has led to the over 200 known CTAs being classified into different families based on primary sequence homology [10]. In many instances, CTA families consist of multiple nearly identical genes that are clustered together, frequently on the X-chromosome [11]. Notably, whole families of CTAs are often co-expressed together in tumors, indicating a shared regulatory mechanism for groups of related CTAs [10]. The frequent reactivation of CTA genes has led to the suggestion that these gametogenic genes functionally participate in conferring neoplastic phenotypes. However, investigations into how CTAs contribute to spermatogenesis or tumor progression have only recently begun to be undertaken. For example, specific CTAs have been implicated in the regulation of centrosome function (CEP55) [12], mitosis (ACRBP) [13], retinoic acid (PRAME) [14] and p53 signaling (MAGEB3) [15]. While these results support the concept that reactivated CTAs can support tumor progression, the extent to which CTA support neoplastic phenotypes, including invasive behavior, remains largely unknown.

Here, we find that SPANX-A/C/D, CTAG2, GAGE and PAGE2-2/B promote breast cancer cell invasion in organotypic culture, revealing that the induction of these CTAs can contribute to the acquisition of neoplastic traits. We further discovered that CTAs have unique sub-cellular distribution patterns and interacting partners, with SPANX-A/C/D forming protein complexes in the inner nuclear membrane and CTAG2 being recruited to the centrosome. Moreover, SPANX-A/C/D was necessary for the formation of protrusions that reorganize the ECM whereas CTAG2 was necessary for directional migration. Thus, the combined re-expression of distinct CTAs influenced unique traits that function together to promote invasive behavior. Importantly, SPANX-A/C/D was necessary for spontaneous metastasis and elevated SPANX-A/C/D expression correlated with poor breast cancer patient outcome, indicating that pro-invasive CTAs can contribute to tumor progression in vivo. Together, these results reveal how the inappropriate induction of gametogenic genes can contribute to neoplastic invasion and metastasis.

## RESULTS

### CTA expression promotes breast cancer invasion

We previously identified an epigenetically distinct subpopulation of “trailblazer” cells that can induce breast cancer collective invasion and form pulmonary metastases (Supplementary Figure S1A–S1C) and [7]. Trailblazer subpopulations were originally isolated manually based on their highly invasive phenotype in organotypic culture and retained their enhanced invasive traits for at least 30 population doublings [7]. In contrast, sibling non-trailblazer cells, termed “opportunist cells”, are not autonomously invasive (Supplementary Figure S1C) and are limited to invading through paths created by trailblazer cells [7]. To uncover anomalously expressed genes that may promote invasion, we evaluated the mRNA content of sibling trailblazer and opportunist subpopulations derived from SUM159 breast cancer cells. Using significance analysis of microarrays (SAM), we determined which genes had at least a 4-fold higher expression in SUM159 trailblazer (SUM159T) cells compared to SUM159 opportunist (SUM159O) cells, with a false-discovery rate (FDR) of 5%. With this approach, we identified 239 probe sets corresponding to 205 genes that were overexpressed in SUM159T cells (Supplementary Table S1). Examination of the classes of genes highly expressed in the trailblazer cells revealed that 28 of the probesets detected gametogenic genes that have been previously categorized as cancer-testis antigens (CTAs), (Table 1). Thus, our expression analysis identified a set of CTAs that are expressed in a highly invasive breast cancer cell population, which suggested that these aberrantly expressed genes had the potential to contribute to the acquisition of invasive traits.

**Table 1:** CTAs are highly expressed in invasive SUM159T cells

CTA family	Probe ID	Genes detected	Fold-change
**GAGE**	1783832	*GAGE*	518.82
	2233576	*GAGE*	491.66
	3244168	*GAGE*	469.63
	3243856	*GAGE*	412.78
	3243856	*GAGE*	337.37
	1664660	*GAGE*	327.03
	3243333	*GAGE*	321.88
	1738450	*GAGE*	316.20
	1715638	*GAGE*	288.43
	3244090	*GAGE*	264.23
	3236963	*GAGE*	262.30
	2104486	*GAGE*	221.07
	3245682	*GAGE*	126.12
	3237846	*GAGE*	104.66
	3242920	*GAGE*	100.21
	3239440	*GAGE*	21.18
	2203976	*GAGE*	18.73
	3236667	*GAGE*	33.59
**PAGE**	1778623	*PAGE1*	115.12
	1724213	*PAGE-2/2B*	254.66
	1706140	*PAGE-2/2B*	336.51
	2363141	*PAGE5*	83.34
**SPANX**	2198300	*SPANX-A/C/D*	12.88
	1680689	*SPANX-B1/B2*	31.99
**CTAG**	2336585	*CTAG2*	23.03
**XAGE**	2190541	*XAGE2B*	32.79
**MAGE**	1688335	*MAGEB2*	74.50
**SPANXN**	1691953	*SPANXN3*	34.29

Fold increase in expression in SUM159T cells (invasive) relative to SUM159O cells (noninvasive). The genes detected by the probes are indicated. Due to the high identity between related CTAs, some probes detect multiple CTA genes. GAGE= GAGE-1/2/4/6/7/8/10/12.

Twenty-five of the 28 probes detecting CTAs corresponded to members of the CTAG [16] GAGE [17], SPANX [18] and PAGE [19] families (Table 1). SPANX, GAGE and PAGE genes are believed to be products of evolutionarily recent duplications of gene located on the X-chromosome, with multiple family member genes being highly similar to each other [20–22]. For example, the GAGE genes have >95% identity with each other [22], SPANX-C shares >95% identity with SPANX-A and SPANX-D [20], and PAGE-2 shares 94% identity with PAGE-2B [23]. As a consequence of this high degree similarity between related genes and the strong potential for functional redundancy, we refer to the cohort of GAGE genes as “GAGE”; SPANX-A, SPANX-B and SPANX-D as “SPANX-A/C/D”; and PAGE-2 and PAGE-2B as “PAGE-2/2B”.

SPANX-A/C/D, CTAG2 and GAGE have been previously identified as CTAs that are induced in breast tumors and are not detected in normal adult female tissues, including mammary epithelium [16, 24, 25]. However, the functional significance of the expression of these CTAs in breast cancer or in the testis has not been explored. Our discovery that PAGE-2/2B was expressed in the SUM159T cells suggested that PAGE-2/2B may function in breast cancer as well. Therefore, to determine the significance of CTA expression, we evaluated the consequence of depleting SPANX-A/C/D, CTAG2, GAGE and PAGE-2/2B on SUM159T invasion.

**Figure 1:**
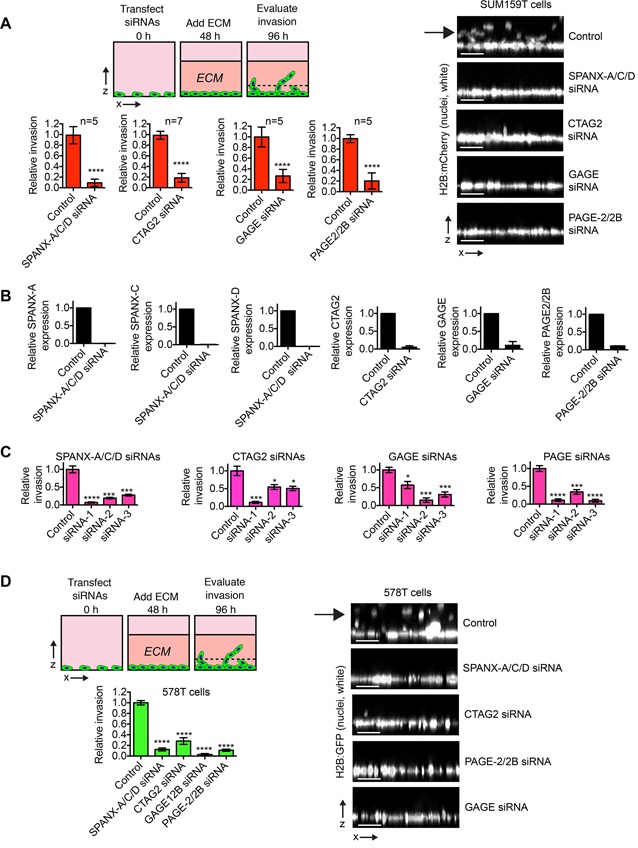
CTA expression promotes breast cancer invasion **A.** Graphs show the relative invasion of transfected SUM159T cells ≥ 30 μm into the ECM. The number of invasive cells is normalized to the total cell number in the field of view for each condition, which controls for any variations in cell number. Relative invasion equals the normalized invasive value of the cells transfected with the CTA targeting siRNA pool divided by the mean normalized invasion value for “control” cells transfected with a pool of 4 siRNAs that do not target human genes. Mean ± standard deviation (SD) of at least 5 independent experiments is shown. The exact number of independent experiments is indicated above each bar graph. ****p< 0.0001, unpaired Student's t test. Images are representative x-z views of SUM159T invasion into ECM after transfection with the indicated pool of siRNAs. The solid black arrow indicates invading cells. Scale bars, 50 μm. **B.** Graphs show relative expression of the indicated CTAs as determined by q-PCR in cells transfected as indicated (mean + range, n=2). **C.** Graphs show the relative invasion of SUM159T cells transfected with individual siRNAs targeting the indicated CTAs ≥30 μm into the ECM. Relative invasion was determined as described for (A). Mean ± SD of 4 biological replicates from 2 independent experiments is shown. * p< 0.05, *** p< 0.001, ****p< 0.0001, unpaired Student's t test. **D.** Graph shows the relative invasion of transfected 578T cells ≥30 μm into the ECM normalized to the total cell number and compared to control cells as described in (A). Mean ± SD of 4 biological replicates from 2 independent experiments is shown. ****p< 0.0001, unpaired Student's t test. Images are representative x-z views of 578T invasion into the ECM after transfection with the indicated siRNAs. The solid black arrow indicates invading cells. Scale bar, 50 μm.

We found that siRNAs targeting SPANX-A/C/D, CTAG2, GAGE and PAGE-2/2B ablated the ability of SUM159T cells to invade into a thick layer of ECM (Figure 1A). Depletion of the CTAs was confirmed by qPCR (Figure 1B), and at least 3 distinct siRNAs targeting each CTA group perturbed invasion (Figure 1C), indicating the observed phenotypes were not attributable to off-target effects. We next tested if SPANX-A/C/D, CTAG2, GAGE and PAGE-2/2B were required for the invasion of 578T cells, which contain a substantial population of trailblazer cells [7]. Indeed, the depletion of CTAG2, GAGE, SPANX-A/C/D and PAGE-2/2B reduced 578T invasion (Figure 1D), indicating that these CTAs are required for invasion in multiple genetic contexts. Together, these results suggest that that anomalous expression of SPANX-A/C/D, CTAG2, GAGE and PAGE-2/2B can contribute to the induction of breast cancer cell invasion.

### SPANX-C interacts with the nucleocytoskeletal proteins Lamin A/C

**Figure 2:**
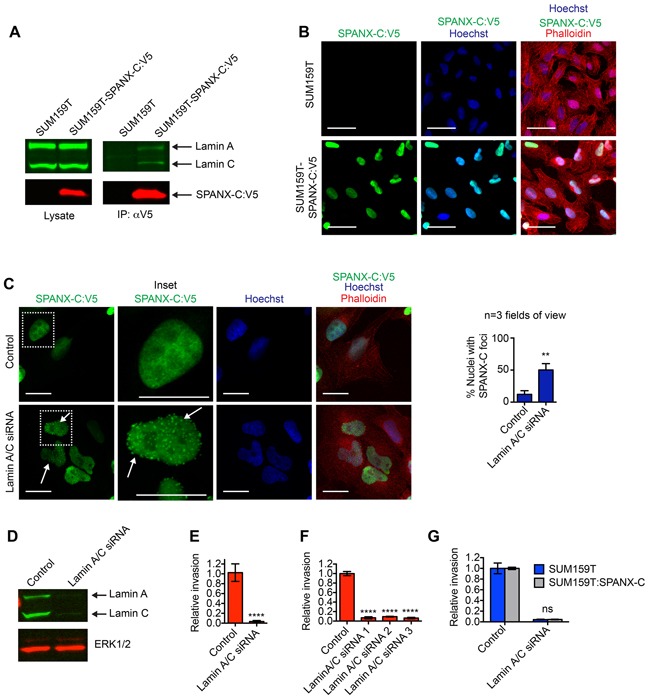
SPANX-A/C/D interacts with Lamin A/C **A.** Cell lysates and associated anti-V5 immunoprecipitates were immunostained with α-V5 and α-Lamin A/C antibodies. Results are representative of 3 independent experiments. **B.** SUM159T and SUM159T-SPANX-C:V5 cells immunostained with α-V5 antibody and counterstained with Hoechst and phalloidin. Results are representative of 3 independent experiments. Scale bars, 50 μm. **C.** SUM159T-SPANX-C:V5 cells transfected as indicated were immunostained with α-V5 antibody and counterstained with Hoechst and phalloidin. Arrows indicate punctate localization of SPANXC:V5. Scale bars, 20 μm. Graph shows the percent of cells with ≥ 2 foci of SPANXC:V5 in the nucleus. Mean+ SD of at least 3 fields of view, each containing ≥35 cells, from 2 independent experiments. **p< 0.01, unpaired Student's t test. **D.** Cell lysates of SUM159T transfected with control or Lamin A/C siRNA pools were immunoblotted with α-Lamin A/C and α-ERK1/2 antibodies. Results are representative of 3 independent experiments. **E.** Graph shows the relative invasion of transfected SUM159T cells ≥ 30 μm into the ECM. The number of invasive cells is normalized to the total cell number in the field of view for each condition, which controls for any variations in cell number. Relative invasion equals the normalized invasive value of the cells transfected with the siRNA pool targeting Lamin A/C divided by the mean normalized invasion for the “control” cells transfected with a pool of 4 siRNAs that do not target human genes. Mean ± standard deviation (SD) of 4 independent experiments is shown. ****p< 0.0001, unpaired Student's t test. **F.** Graph shows the relative invasion of SUM159T cells transfected with individual siRNAs targeting Lamin A/C ≥30 μm into the ECM. Relative invasion was determined as described for (E). Mean ± standard error of the mean (SEM) of 4 biological replicates from 2 independent experiments is shown. ****p< 0.0001, unpaired Student's t test. **G.** Graph shows the relative invasion of SUM159T and SUM159T-SPANX-C cells transfected with control or Lamin A/C targeting siRNAs ≥30 μm into the ECM. Relative invasion was determined as described for (E). Mean ± standard error of the mean (SEM) of 4 biological replicates from 2 independent experiments is shown. ns= not significant, unpaired Student's t test.

SPANX-A/C/D has been detected in metastatic melanoma cells [18] and increased SPANX-A/C/D expression correlates with liver metastasis in colorectal cancer patients [26]. Whether the expression of SPANX-A/C/D was causal in supporting metastasis or epiphenomenon was not determined. Given the potential significance of SPANX-A/C/D invasion across multiple tumor types, we further investigated SPANX-A/C/D function. SPANX-A/C/D proteins are 97 amino acids in length and do not have predicated enzymatic functions, suggesting that SPANX-A/C/D may influence cell behavior through interactions with other proteins. Therefore, to begin determining how SPANX-A/C/D promoted invasion, we identified proteins that were co-immunoprecipitated with a SPANX-C-V5 fusion protein by mass spectrometry. We found that there was an enrichment for peptide sequences corresponding to products of the LMNA gene (Lamin A and Lamin C, herein referred to as Lamin A/C) in the SPANX-C-V5 immunoprecipitated samples (Supplementary Table S2). This interaction between SPANX-C and Lamin A/C was confirmed by immunoblotting of SPANX-C immunoprecipitates with α-Lamin A/C antibody (Figure 2A). Lamin A/C are components of a meshwork of nuclear lamina proteins that underly the inner nuclear membrane and provide structural stability to the nucleus [27]. Indeed, consistent with the detection of Lamin-A/C in SPANX-C protein complexes, we found that SPANX-C was localized to the nucleus of SUM159T cells (Figure 2B). To determine if the interaction between SPANX-C and Lamin A/C influenced SPANX-C localization, we evaluated the subcellular distribution of SPANX-C in Lamin A/C depleted cells. We found that SPANX-C was retained in the nucleus of Lamin A/C depleted SUM159T cells, possibly due to the presence of a putative nuclear localization signal in the SPANX-C protein [18]. However, a fraction of the SPANX-C became re-localized into punctate foci indicating that the loss of Lamin A/C expression altered how SPANX-C was distributed within the nucleus (Figure 2C and 2D). Together, these results suggest that the interaction between SPANX-C and Lamin A/C is necessary for proper SPANX-C localization.

Our results characterizing the sub-cellular distribution and protein interaction partners of SPANX-C, suggested that SPANX-C may regulate functions within the nucleus that are required for invasive behavior. To determine if the interaction between SPANX-C and Lamin A/C could potentially influence invasive traits, we determined how depletion of Lamin A/C influenced SUM159T invasion. Transfection of SUM159T cells with Lamin A/C siRNAs reduced invasion into the ECM (Figure 2E). Similar results were observed with 4 different siRNAs, indicating that the suppression of invasion was a consequence of Lamin A/C depletion (Figure 2F). Exogenous SPANX-C was not able to restore invasive ability in Lamin A/C depleted cells (Figure 2G), which is consistent with Lamin A/C being necessary to properly localize SPANX-C. Lamin A/C may also regulate other essential pro-invasive functions, such as anchoring the nucleus to the cytoskeleton by forming the Linker of the Nucleus to the Cytoskeleton (LINC) complexes [28, 29]. Together, these results indicate that SPANX-C interacts with structural proteins that are necessary for invasion.

### SPANX-A/C/D and Lamin A/C are necessary for the formation of cellular protrusions

**Figure 3:**
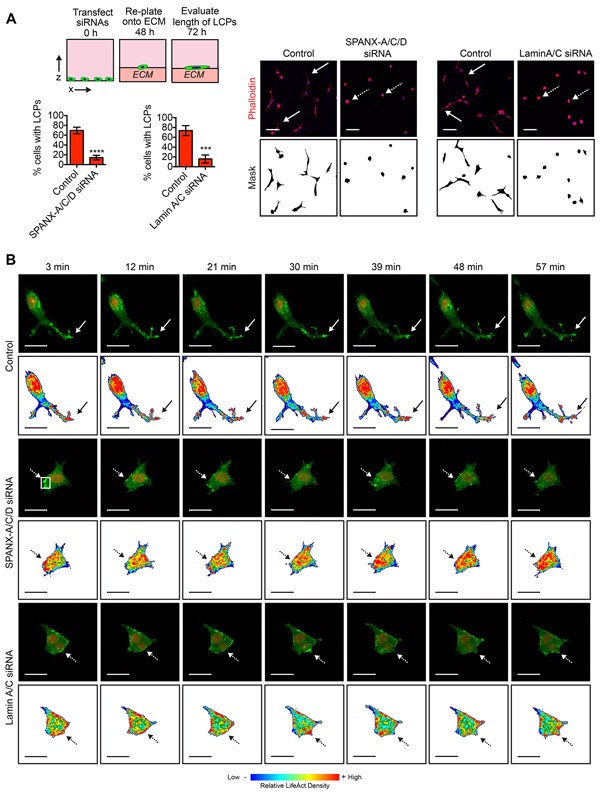
SPANX-A/C/D and Lamin A/C are specifically required for LCP formation **A.** SUM159T cells transfected with control, SPANX-A/C/D or Lamin A/C siRNA pools were plated onto a layer of ECM for 24 h and stained. Solid white arrows indicate representative cells with LCPs. Dashed white arrows indicate representative cells that fail to form LCPs. Scale bar, 100 μm. Graphs show the percentage of cells with a l/w ratio ≥2, indicating LCP formation. Mean± SD of 6 fields of view, each containing ≥40 cells, from 3 independent experiments for SPANX-A/C/D siRNA transfected cells. Mean+ SD of 4 fields of view, each containing ≥40 cells, from 2 independent experiments for Lamin A/C siRNA transfected cells. ***p< 0.001, ****p< 0.0001, unpaired Student's t test. **B.** Time lapse images of the SUM159T-LifeACT:GFP-H2B:mCherry cells transfected with control, SPANX-A/C/D or Lamin A/C siRNA pools. Representative images of 16 cells imaged over 2 independent experiments are shown. Also see Supplementary Video S1. The top panels for each condition show LifeACT:GFP (green) and H2B:mCherry (red). Bottom panels for each condition show heat maps depicting relative LifeACT:GFP signal intensity with red indicating strongest signal. Solid arrows identify a representative LCP. Dashed arrows identify regions where F-actin formation is dynamic. Scale bar, 25 μm.

During collective invasion, leading tumor cells form long cellular protrusions (LCPs) that reorganize the ECM to facilitate cell translocation through a confined space [30]. The requirement of SPANX-A/C/D and Lamin A/C for SUM159T invasion in organotypic culture suggested that these genes may contribute to the formation of LCPs. To test this possibility, we evaluated LCP formation in SUM159T cells transfected with siRNAs targeting SPANX-A/C/D and Lamin A/C. Indeed, depletion of SPANX-A/C/D and Lamin A/C reduced the percentage of cells that formed LCPs (Figure 3A).

To determine how actin dynamics and protrusive behavior were influenced by SPANX-A/C/D depletion, we performed time-lapse imaging on SUM159T cells expressing LifeACT:GFP, which specifically localizes to F-actin [31]. In the control SUM159T cells, we detected dynamic changes in LifeACT:GFP signal intensity, indicating that there was formation and severing of F-actin around the surface of the cell. Notably, we found that there was an enrichment of LifeACT:GFP in the tips of the LCPs, indicating that F-actin formation contributed to protrusion formation. (Figure 3B and Supplementary Video S1). Moreover, the tips of protrusions were dynamic, both extending and retracting (Figure 3B and Supplementary Video S1), consistent with LCPs forming attachments and physically reorganizing ECM fibers to facilitate cell translocation. However, the overall length of the protrusions was not significantly reduced. In SPANX-A/C/D and Lamin A/C depleted cells, the actin cytoskeleton was also dynamic, as reflected by changes in the intensity of LifeACT:GFP fluorescent signal around the surface of the cells. Importantly, SPANX-A/C/D and Lamin A/C depleted cells formed transient protrusions containing LifeACT:GFP (Figure 3B and Supplementary Video S1). However, in contrast to the control SUM159T cells, the protrusions in the SPANX-A/C/D or Lamin A/C depleted cells did not extend to form LCPs (Figure 3B and Supplementary Video S1). Together, our results indicate that the SPANX-A/C/D and Lamin A/C are specifically required for the induction of LCPs. However, SPANX-A/C/D and Lamin A/C are not necessary for the formation and severing of F-actin or the induction of small transient actin-rich protrusions.

### SPANX-C expression is not sufficient to induce invasion

**Figure 4:**
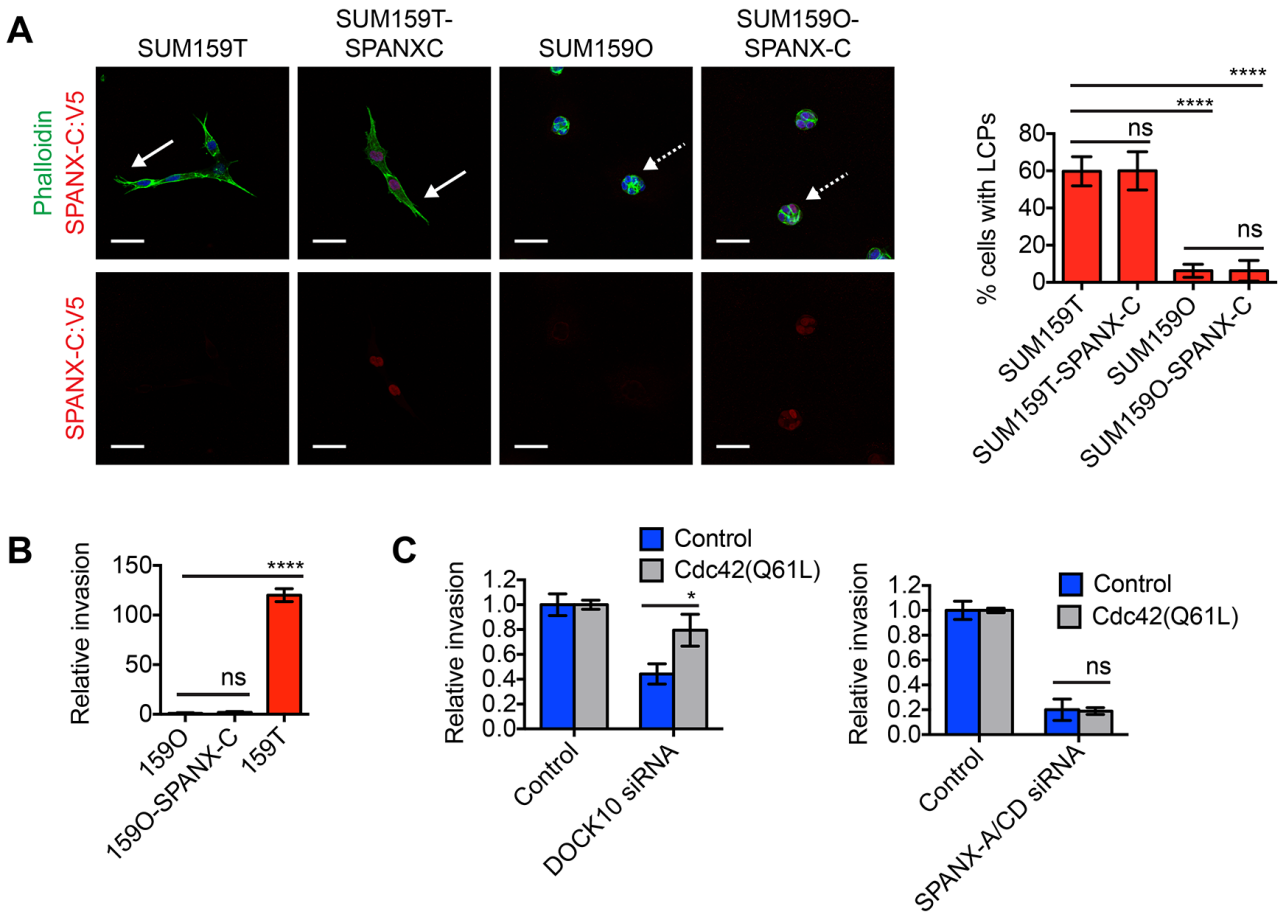
SPANX-C is not sufficient to induce LCP formation or collective invasion **A.** SUM159T, SUM159T-SPANXC:V5, SUM159O and SUM159O-SPANXC:V5 cells were plated onto a layer of ECM for 24 h. Cells were then fixed and immunostained with α-V5 antibody and counterstained with Hoechst and phalloidin. Solid white arrows indicate representative cells with LCPs. Dashed white arrows indicate representative cells that fail to form LCPs. Scale bar, 50 μm. Graph shows the percentage of cells with a l/w ratio ≥2, indicating LCP formation. Mean± SD of 4 fields of view, each containing ≥40 cells, from 2 independent experiments. ****p< 0.0001, ns= not significant, unpaired Student's t test. **B.** Graph shows the relative invasion of cells ≥30 μm into the ECM normalized to the total cell number and compared to SUM159O cells. Mean ± SD of 4 biological replicates from 2 independent experiments is shown. ns= not significant, ****p< 0.0001, unpaired Student's t test. **C.** Graphs show the relative invasion of SUM159T and SUM159T-Cdc42(Q61L) cells ≥30 μm into the ECM after transfection with control, DOCK10 or SPANX-A/C/D siRNA pools. Mean ± SEM of 4 biological replicates from 2 independent experiments is shown. *p< 0.05, ns= not significant, unpaired Student's t test.

Our results showing that SPANX-A/C/D was essential for LCP formation and invasion, combined with our expression analysis showing elevated SPANX-A/C/D levels in SUM159T cells, suggested that SPANX-A/C/D overexpression may be sufficient to confer cells with an invasive phenotype. To test this possibility, we measured the capacity of exogenous SPANX-C to induce the invasion of SUM159O cells, which are the relatively noninvasive compared to their sibling SUM159T cells (Supplementary Figure S1C) and [7]. Exogenous SPANX-C localized to the nucleus of SUM159O cells, indicating that SPANX-A/C/D was correctly localized to influence invasive behavior (Figure 4A). However, overexpressed SPANX-C was unable to induce LCP formation (Figure 4A) or invasion into the ECM (Figure 4B). The inability of SPANX-C to induce SUM159O invasion is likely due to that fact that SUM159O cells do not express the other invasion promoting CTAs, as well as other genes induced in SUM159T cells, including DOCK10, DAB2, α11 integrin and PDGFRA [7].

The expression of a constitutively active variant of Cdc42 (Cdc42 (Q61L)) is sufficient to restore invasive ability in DOCK10 depleted SUM159T cells, but not PDGFRA and DAB2 depleted cells [7]. These results indicate that both Cdc42-dependent and Cdc42-independent signaling pathways are necessary for SUM159T invasion. To determine if SPANX-A/C/D induced invasion through the regulation of Cdc42, we tested if Cdc42(Q61L) was sufficient to restore invasive ability in SPANX-A/C/D depleted cells. Similar to our previous findings, we found that Cdc42(Q61L) enhanced the invasion of SUM159T cells depleted of DOCK10 (Figure 4C). In contrast, Cdc42(Q61L) failed to restore invasive ability in SUM159T cells depleted of SPANX-A/C/D (Figure 4C), indicating that SPANX-A/C/D primarily promotes invasion through the activation of a Cdc42-independent signaling pathway. Together, our observations indicate that SPANX-A/C/D functions are integrated together with additional signaling pathways regulated by DOCK10, DAB2 and ITGA11 to promote LCP formation and invasion.

### CTAG2 is necessary for directional migration

**Figure 5:**
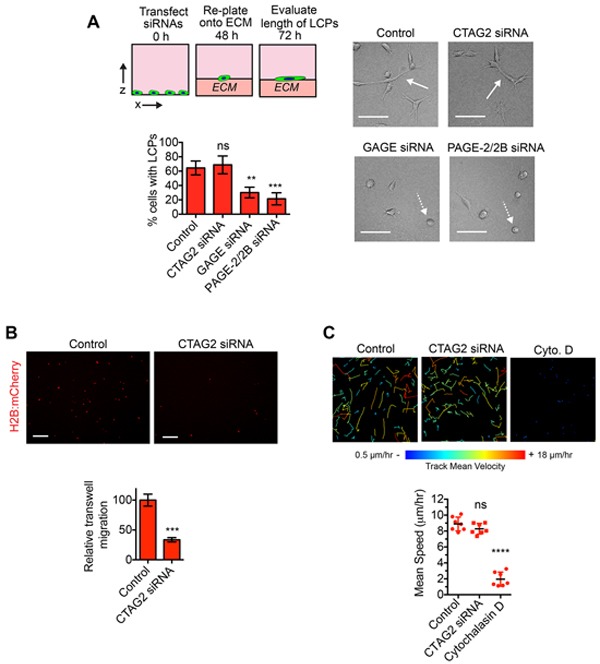
CTAG2 is necessary for directional migration **A.** SUM159T cells transfected with the indicated siRNAs were plated onto a layer of ECM for 24 h. Solid white arrows indicate representative cells with LCPs. Dashed white arrows indicate representative cells that fail to form LCPs. Scale bar, 100 μm. Graph shows the percentage of cells with a l/w ratio ≥2, indicating LCP formation. Mean± SD of 4 fields of view, each containing ≥40 cells, from 2 independent experiments. ns= not significant, **p< 0.01, ***p< 0.001, unpaired Student's t test. **B.** SUM159T-H2B:mCherry cells were transfected with control or CTAG2 siRNA pools for 48 h and then re-plated onto transwell inserts in serum free media. Transfected cells were then allowed to migrate towards serum containing media for 24 h. Representative images of SUM159T-H2B:mCherry cells that migrated to the underside of the transwell insert are shown. Scale bar, 100 μm. Graph shows the number of cells that migrated through the insert divided by the total number of cells on the top and underside of the insert and normalized to the control. Mean ± SEM of 4 independent experiments. ***p< 0.001, unpaired Student's t test. **C.** The movement of SUM159T-H2B:mCherry cells transfected with control or CTAG2 siRNAs over a 14 h time period in monolayer culture. The color indicates the speed of cell movement in the track. Scale bars, 50 μm. Graph shows the mean cell speed (mean ±SD, n=7 fields of view over 2 independent experiments). ****p< 0.0001, ns= not significant, unpaired Student's t test.

Our results demonstrating that SPANX-A/C/D was necessary for LCP formation suggested that CTAG2, GAGE and PAGE-2/2B may also participate in LCP regulation. Indeed, depletion of GAGE and PAGE2/2B reduced the percentage of SUM159T cells that formed LCPs (Figure 5A), indicating that multiple distinct CTA families can contribute to LCP induction. However, CTAG2 depletion did not detectably alter the percentage of LCP forming cells (Figure 5A), indicating that CTAG2 contributed to some other aspect of invasive behavior. To determine if CTAG2 was necessary for directional migration, we quantified the motility of SUM159T cells towards serum in a transwell migration assay. CTAG2 depletion reduced the number of cells migrating towards serum through a transwell filter (Figure 5B), suggesting that CTAG2 may be required for directional sensing during invasion. By comparison, CTAG2 was not required for the spontaneous movement of SUM159T cells in monolayer culture (Figure 5C), indicating that CTAG2 was not broadly required for all forms of cell movement. Together, these results suggest that CTA families have distinct essential functions that are integrated together to promote collective invasion.

### CTAG2 interacts with the centrosomal protein Pericentrin

**Figure 6:**
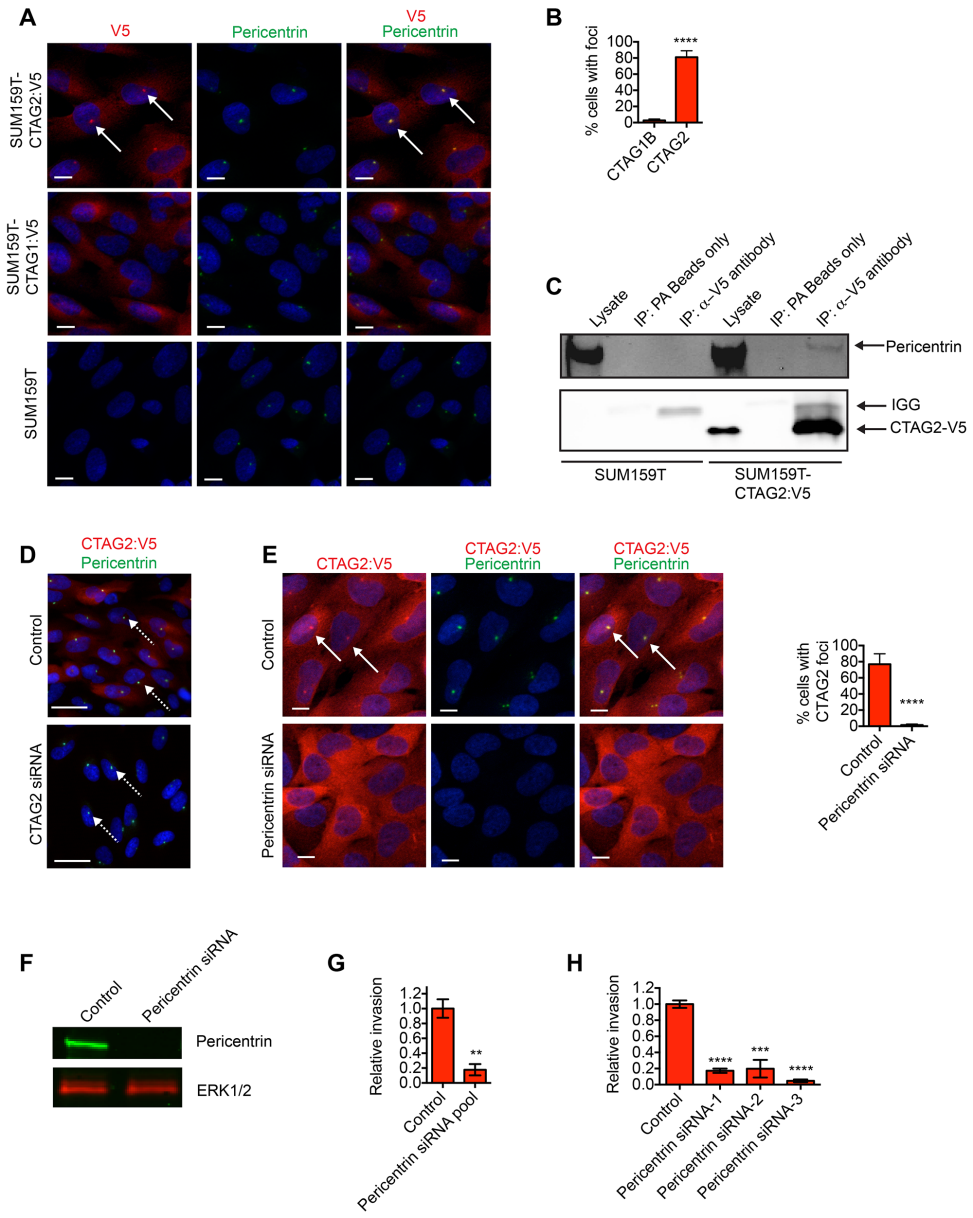
CTAG2 interacts with Pericentrin **A.** SUM159T, SUM159T-CTAG2:V5 and SUM159T-CTAG1B:V5 cells immunostained with α-V5 and α-Pericentrin antibodies and counterstained with Hoechst. Scale bar, 10 μm. White arrows indicate representative CTAG:V5 foci that co-localize with Pericentrin. **B.** Graph shows percentage of cells with foci of CTAG1B or CTAG2 that co-localize with Pericentrin. Mean± SD of 4 fields of view containing at least 20 cells from 2 independent experiments. ****p< 0.0001, unpaired Student's t test. **C.** Cell lysates and associated α-V5 immunoprecipitates were immunoblotted with α-V5 and α-Pericentrin antibodies. Lysates incubated with protein A sepharose (PA) beads are included as a negative control. Results are representative of 3 independent experiments. **D.** SUM159T-CTAG2:V5 cells transfected as indicated were immunostained with α-V5 and α-Pericentrin antibodies and counterstained with Hoechst. Dashed white arrows indicate punctate localization of Pericentrin. Scale bar, 25 μm. **E.** SUM159T-CTAG2:V5 cells transfected as indicated were immunostained with α-V5 and α-Pericentrin antibodies and counterstained with Hoechst. White arrows indicate representative CTAG:V5 foci that co-localize with Pericentrin. Scale bar, 10 μm. Graph shows percentage of cells with foci of CTAG2 that co-localize with Pericentrin. Mean± SD of 4 fields of view containing at least 20 cells from 2 independent experiments. ****p< 0.0001, unpaired Student's t test. **F.** Cells lysates of SUM159T transfected with control or Lamin A/C siRNA pools were immunoblotted with α-Pericentrin and α-ERK1/2 antibodies. Results are representative of 3 independent experiments. **G.** Graph shows the relative invasion of SUM159T cells transfected with control or Pericentrin siRNA pools ≥ 30 μm into the ECM. The number of invasive cells is normalized to the total cell number in the field of view for each condition, which controls for any variations in cell number. Relative invasion equals the normalized invasive value of the cells transfected with the siRNA pool targeting Pericentrin divided by the mean normalized invasion for the “control” cells transfected with a pool of 4 siRNAs that do not target human genes. Mean ± SEM of 3 independent experiments is shown. ****p< 0.0001, unpaired Student's t test. **H.** Graph shows the relative invasion of SUM159T cells transfected with individual siRNAs targeting Pericentrin ≥30 μm into the ECM. Relative invasion was determined as described for (G). Mean ± SEM of 4 biological replicates from 2 independent experiments is shown. *** p< 0.001, ****p< 0.0001, unpaired Student's t test.

Given the unique contribution of CTAG2 in promoting invasion compared to SPANX-A/C/D, GAGE and PAGE2/2B, as well as observations that CTAG2 is frequently reactivated in estrogen receptor negative (ER-neg) patient tumors [32], we next investigated CTAG2 sub-cellular distribution. Consistent with their different functions during invasion, the subcellular distribution of CTAG2 was distinct from SPANX-A/C/D, with CTAG2 distributed throughout the cell (Figure 6A). Notably, a fraction of CTAG2 was highly enriched in a single punctate focus near the nucleus (Figure 6A and 6B), which was reminiscent of the organization of proteins that form the centrosome [33]. To determine if CTAG2 was indeed localized to the centrosome, we examined the interaction between CTAG2 and Pericentrin, a scaffolding protein which is a component of the pericentriolar material (PCM) that surrounds the centrioles [34]. We found that CTAG2 co-localized with Pericentrin in SUM159T cells (Figure 6A and 6B), and that Pericentrin co-immunoprecipitated with CTAG2 (Figure 6C). By comparison, the related CTA gene CTAG1B, which is expressed in SUM159O and SUM159T cells, did not co-localize with Pericentrin (Figure 6A and 6B) and was not required for SUM159T invasion (Supplementary Figure S2A and S2B). Pericentrin is transported to the centrosome along microtubules as part of a multi-protein complex that includes Centrin, Ninein and PCM1 [35, 36]. To determine if CTAG2 was necessary for the centrosomal recruitment of Pericentrin, we examined the localization of Pericentrin in CTAG2 depleted SUM159T. CTAG2 depletion did not alter the translocation of Pericentrin to the centrosome, as indicated by the sustained punctate localization of Pericentrin (Figure 6D). Thus, CTAG2 was not required for Pericentrin transport. However, Pericentrin depletion did ablate the foci of CTAG2 (Figure 6E and 6F), indicating that CTAG2 physically interacts with the centrosome in intact cells.

Pericentrin has been implicated as a microtubule nucleation factor that is necessary for the centrosome to function as the microtubule organizing center (MTOC) of cells [34]. Microtubules can contribute to migration by defining cell polarity, regulating cell substrate adhesion and directing the intracellular transport of essential motility regulatory factors [37]. In addition, increased microtubule polymerization resulting from centrosome amplification can promote invasion [38]. Therefore, we determined how depletion of Pericentrin influenced the invasive ability of SUM159T cells. Depletion of Pericentrin reduced SUM159T invasion (Figure 6G), with similar results observed with 3 unique siRNAs (Figure 6H). Together, these results indicate that CTAG2 interacts with a protein complex containing Pericentrin that is necessary for invasive behavior.

### CTA expression is necessary for primary tumor growth and pulmonary metastasis

**Figure 7:**
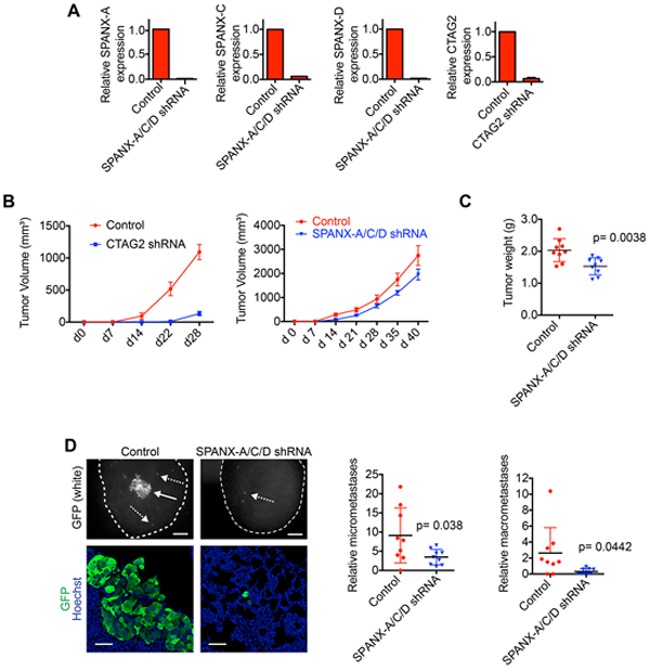
SPANX-A/C/D is required for metastasis **A.** Relative expression of the indicated CTAs in SUM159T-GFP cells stably expressing shRNAs targeting SPANX-A/C/D or CTAG2 (n=2 mean ± range). **B.** Graphs show the volumes of tumors formed by SUM159T cells expressing control, CTAG2 and SPANX-A/C/D shRNAs (mean ± SD, n=9 mice/condition). **C.** Graph shows the weights of tumors formed by SUM159T cells expressing control and SPANX-A/C/D shRNAs (mean ± SD, n=9 mice/condition). P-values were determined by unpaired Student's t test. **D.** Representative fluorescent images of lungs from mice bearing control and SPANX-A/C/D shRNA expressing SUM159T-GFP primary tumors. Top panels, GFP expression of SUM159T-GFP cells in the lungs immediately after mice were sacrificed (n=9 mice/condition). Solid arrow indicates a macrometastic lesion. Dashed arrows indicate representative micrometastases. Scale bars, 200 μm. Bottom panels show representative images of lungs immunostained with α-GFP antibody and counterstained with Hoechst (n=9 mice/condition). Scale bars, 50 μm. Graphs show the relative number of micrometastases (left) and macrometastases (right) in the in the lungs normalized to the weight of the corresponding primary tumor (n=9 mice/condition). P-values determined by unpaired Student's t test.

Our results demonstrating that SPANX-A/C/D and CTAG2 were necessary for invasion suggested that these CTAs may contribute to tumor development in vivo. To test this possibility, we determined the characteristics of tumors formed by SUM159T cells depleted of SPANX-A/C/D or CTAG2, (Figure 7A). CTAG2 depletion drastically reduced primary tumor growth (Figure 7B), indicating that CTAG2 regulates additional functions related to tumor growth. We did not detect a similar reduction in the number of CTAG2 depleted cells in the monolayers of our invasion assay (Supplementary Figure S2C). Unlike CTAG2, SPANX-A/C/D depletion had a relatively small impact on primary tumor growth (Figure 7B and 7C), indicating that the growth dependency observed for CTAG2 was not a general feature of CTAs expressed in SUM159T cells. Importantly, SPANX-A/C/D depletion reduced the number of single cell micrometastases and multicellular macrometastases in the lungs compared to mice bearing control SUM159T primary tumors (Figure 7D), even when controlling for any differences in primary tumor size. These results indicate that SPANX-A/C/D dependent regulation of invasion may contribute to the dissemination of tumor cells to distant tissues. It is also possible that SPANX-A/C/D contributes to additional processes required for metastasis, such as the extravasation of cells from blood vessels. Together, our results indicate that SPANX-A/C/D is required the formation of pulmonary metastases.

### SPANX-A/C/D expression correlates with poor patient outcome

**Figure 8:**
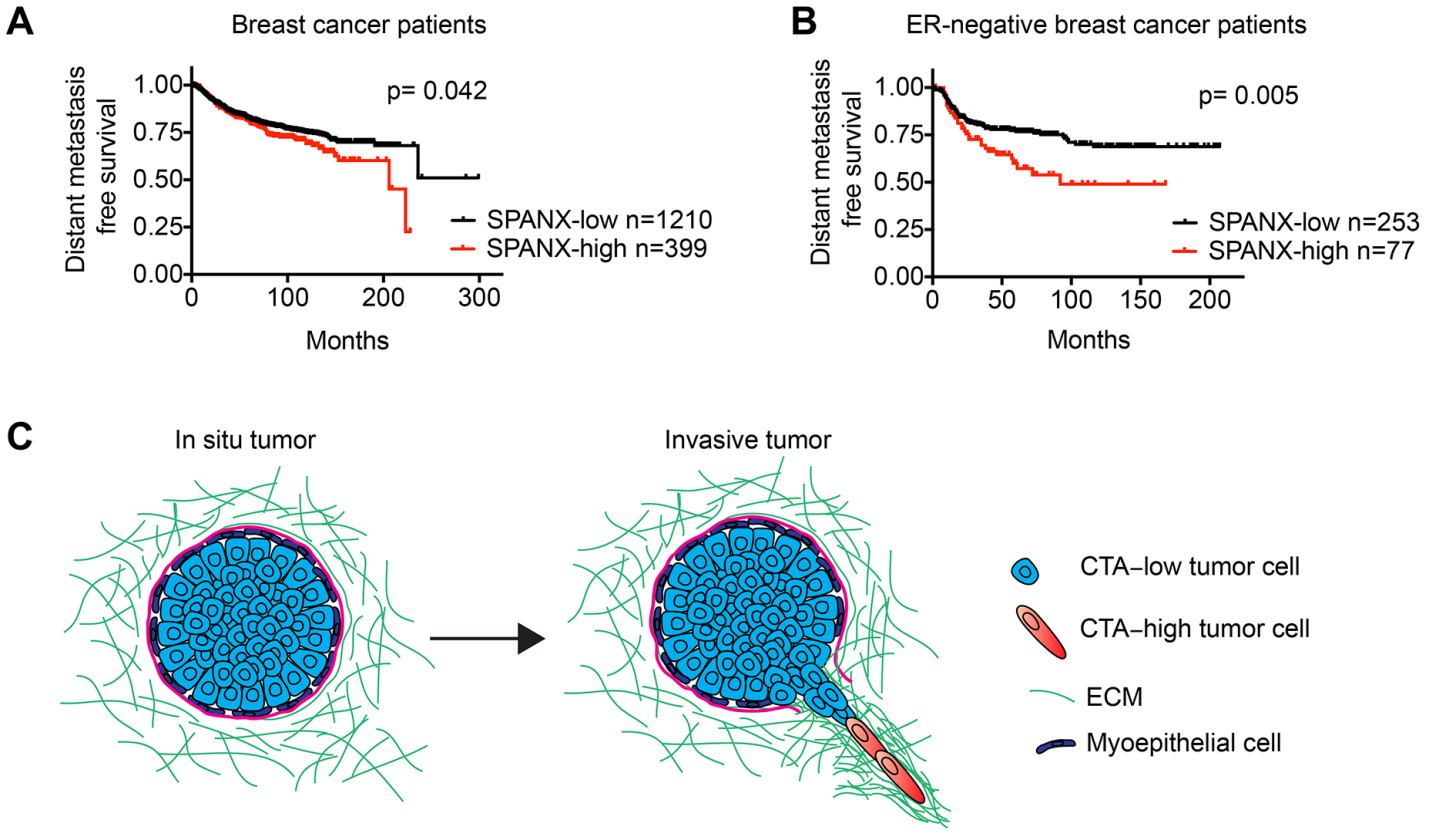
Increased SPANX-A/C/D expression correlates with poor breast cancer patient outcome **A.** Kaplan-Meier curves showing the distant metastasis free survival of breast cancer patients classified as “SPANX-A/C/D high” and “SPANX-A/C/D low” based on SPANX-A/C/D mRNA expression. Survival differences were compared by log-rank test. **B.** Kaplan-Meier curves showing the distant metastasis free survival of ER-neg patients classified as “SPANX-A/C/D-high” and “SPANX-A/C/D-low” based on SPANX-A/C/D mRNA expression. Survival differences were compared by log-rank test. Analysis of publicly available data sets was performed using KM-Plotter. **C.** Model showing how the induction of CTA expression could possibly contribute to the initiation of tumor invasion. Cells expressing CTAs can extend LCPs and migrate into the ECM surrounding primary tumors.

In light of our discovery that SPANX-A/C/D was necessary for invasion and metastasis, we next determined how SPANX-A/C/D expression correlated with distant metastasis free survival in breast cancer patients. High SPANX-A/C/D expression correlated with an increased odds of distant metastasis detection when evaluating all breast cancer patients in the cohort (Figure 8A). Notably, an even stronger correlation between increased SPANX-A/C/D expression and shorter distant metastasis free survival time was observed in ER-neg patients (Figure 8B). These clinical observations are consistent with our results showing that SPANX-A/C/D can promote the invasion and metastasis of ER-neg breast cancer cells. Thus, SPANX-A/C/D warrants further investigation as a biomarker for increased risk of recurrent metastatic disease.

## DISCUSSION

Through a combination of gene expression analysis and function testing, we found that the CTA genes SPANX-A/C/D, CTAG2, GAGE and PAGE-2/2B can be essential for breast cancer invasion (Figure 8C). Like the majority of CTAs, it was unknown whether SPANX-A/C/D, CTAG2 and PAGE-2/2B supported neoplastic traits. Thus, our discovery that SPANX-A/C/D, CTAG2, PAGE-2/2B can be necessary for invasion reveals a mechanistic contribution for these CTAs during tumor progression. Importantly, SPANX-C and CTAG2 are also expressed in prostate, lung, bladder, sarcoma and melanoma cancers [16, 24], suggesting that these genes may promote invasion in additional tumor types. Our results revealing that GAGE controls breast cancer invasion extends on prior findings implicating GAGE in the regulation of melanoma cell migration [39] and gastric cancer metastasis [40]. These previous results, along with our new findings, suggest that GAGE may also be necessary for the invasion of bladder and lung tumors, which are known to express GAGE [25, 41].

In determining how CTAs promote invasion, we discovered that SPANX-A/C/D was necessary for the sustained extension of actin-containing LCPs, which are detected in the trailblazer cells leading invasion into the ECM. Conversely, SPANX-A/C/D was dispensable for the induction of transient protrusions and F-actin filament formation and severing. Therefore, our findings indicate that SPANX-A/C/D was specifically required for the formation of LCPs, but not generally required for the regulation of the actin cytoskeleton. SPANX-A/C/D localizes to the nucleus and is incorporated into protein complexes that contain the nucleoskeletal proteins Lamin A/C, consistent with previous observations that SPANX-A and SPANX-C localize to the nucleus in sperm [24, 42]. However, sperm do not form LCPs. Thus, our results suggest that SPANX-A/C/D dependent regulation of LCPs is a neomorphic function.

Nuclei can be required to undergo deformation when individual tumor cells migrate through confined spaces, such as the ECM in the tumor microenvironment [43]. However, the formation of LCPs by cells plated on the surface of the ECM does not require cells to enter a confined space. Therefore, our results indicate that SPANX-A/C/D does not promote LCP induction by reducing nuclear stiffness. SPANX-C interacts with Lamin A/C, which regulates gene expression by scaffolding the formation of multi-protein complexes that modulate chromatin accessibility [44] and influence the ability of transcription factors to interact with DNA [45]. For example, Lamin A/C forms a complex with the LEM-domain containing protein Emerin and barrier-to-autointegration factor (BAF) to tether chromatin to the inner nuclear membrane and silence gene expression [46, 47]. Lamin A/C-Emerin complexes also suppress gene expression by directly interacting with the transcription factors β-catenin [48] and Lmo7 [49]. Thus, in principle, SPANX-A/C/D could contribute to invasion by modulating gene expression through interacting with Lamin A/C-Emerin, or other Lamin A/C-LEM domain containing protein complexes. SPANX-A/C/D could also influence gene expression by altering the characteristics of the LINC complexes that transduce mechanical signals to Lamin A/C-Emerin [28]. Together, our results suggest that further delineating the function of SPANX-A/C/D may provide critical insight into how regulatory functions in the nucleus promote invasive behavior.

Our results suggest that CTAG2 can promote the directional migration of breast cancer cells. While most CTAs are only expressed in primates [11], the C-terminal 100 amino acids of CTAG2 shares a 52% conservation in sequence with the S. cerevisiae protein Pcc1 [50]. Structural studies have revealed that Pcc1 forms an anti-parallel dimer, which interacts with the Kae1 endopeptidase, the Bud32 kinase and Cgi121 to form the KEOPs complex [51]. These findings demonstrate that the PCC1 domain is capable of promoting self-association and can direct interactions with other proteins. Thus, our results suggest that CTAG2 contributes to invasion through regulating protein-protein interactions. Consistent with this possibility, we have found that a portion of CTAG2 protein localizes to the centrosome and interacts with the scaffolding protein Pericentrin. CTAG2 could potentially influence the repositioning of the centrosome, the regulation of microtubule dynamics, or intracellular trafficking along microtubules, all of which can contribute to the establishment of cell polarity and directional migration [37]. CTAG2 could accomplish these tasks by influencing the stability of protein-protein complexes found at centrosomes, or possibly by recruiting proteins not normally found at the centrosomes of somatic cells. CTAG2 may also have additional functions, since it is distributed throughout the cell, possibly reflecting a function of CTAG2 in the transport of cargo along microtubules. Thus, our results suggest a key role for the centrosome in promoting collective invasion, and suggest that this function of the centrosome may be influenced by the re-expression of CTAG2.

CTA levels were increased in cells that have converted to an invasive trailblazer state. We have previously determined that the induction of the trailblazer phenotype involves an epigenetic conversion in cell state that results in the acquisition of heritable invasive traits, such as the ability to form LCPs [7]. CTAs are epigenetically silenced in normal tissue and often induced in tumor cells by a reduction in DNA methylation [10]. Thus, our results suggest that CTA expression can be increased in trailblazer cells as part of a larger alteration in epigenetic state that includes the decreased methylation of CpG islands in the regulatory regions of CTAs. In addition, CTAs are often focally expressed in subpopulations of tumor cells, consistent with our previous findings that the trailblazer cells can be less than 10% of the total tumor cell population [7]. Importantly, trailblazer cells are capable of inducing the opportunistic invasion of ductal carcinoma in situ cells in an orthotopic tumor model [7]. Thus, our results suggest a potential mechanism for how de-repression of CTA expression in a subpopulation of tumor cells could contribute to the transition from benign to malignant tumor growth (Figure 8C).

Consistent with our previous study [7], we found that SUM159T primary tumor cells can metastasize to the lung. We did not detect SUM159T micrometastases or macrometastases in other tissues using GFP fluorescence. Whether this inability to detect SUM159T cells in other frequent sites of breast cancer metastasis, such as the brain or bones [1], reflects an intrinsic preference of trailblazer cells for growth in pulmonary tissue relative to other locations, similar to the distinct tropisms of clonal MDAMB231 breast cancer cell subpopulations [52], is not yet known. Our results here, and previously [7], demonstrate that SPANX-A/C/D and the Cdc42 activating guanine nucleotide exchange factor DOCK10 are required for trailblazer cells to form of LCPs and for SUM159T cells to metastasize to the lung. Our results showing that a constitutively active mutant of Cdc42 sustains the invasive ability of DOCK10 depleted SUM159T cells, but not SPANX-A/C/D depleted SUM159T cells, indicates that SPANX-A/C/D regulates a Cdc42 independent signaling pathway. Together, these findings suggest that SPANX-A/C/D and DOCK10 may cooperate to promote metastasis through the induction of LCPs and collective invasion. Further investigation is necessary to determine whether SPANX-A/C/D or DOCK10 can also contribute to other key processes, such as intravasation and extravasation of tumor cells from blood vessels [1].

Trailblazer cells are a subset of a larger cohort of cells with mesenchymal features in breast cancer cell populations [7]. However, it is not known if canonical traits of EMT programs, such as the loss of epithelial character, the expression of EMT promoting transcription factors or the induction of mesenchymal cytoskeletal proteins [53], are necessary for trailblazer cell invasion. Similarly, it has not been determined if EMT regulatory processes influence the epigenetic regulation of SPANX-A/C/D or CTAG2. It has recently been shown that metastasis can occur without the activation of an EMT program in genetically engineered mouse models of breast and pancreatic cancer [54, 55]. Whether the induction of pro-invasive CTA expression and the conversion to the trailblazer state represents an EMT independent mechanism for conferring invasive ability is an interesting line of future investigation.

In summary, we have found that the induction of genes that are normally restricted to expression in the testis can be essential for the invasive behavior of breast tumor cells. Because CTAs are not expressed in adult female tissue, CTAs, or CTA-regulated processes may be ideal intervention points for anti-metastatic therapies designed to thwart neoplastic invasion and metastasis.

## MATERIALS AND METHODS

### Cell culture and reagents

SUM159 cells were cultured in Ham's F-12 medium containing 5% fetal bovine serum (FBS, Hyclone), 1x penicillin streptomycin solution (Hyclone), 5 μg/ml insulin (Sigma Aldrich), and 1mg/ml hydrocortisone (Sigma-Aldrich). 578T cells (gift from Michael Peyton, Adi Gazdar and John Minna, UTSW) were cultured in a base medium of RPMI (Hyclone), 10% fetal bovine serum (FBS, Hyclone) and 1x penicillin streptomycin solution (Hyclone). Cell lines were validated by Powerplex genotyping within 6 months of use. SUM159T cells stably expressing LifeACT-GFP and PGK-H2B:mCherry were generated as described [56]. Growth factor reduced Matrigel (BD Biosciences, 10-12 mg/ml stock concentration) and bovine dermal collagen I (BD Biosciences) were used for organotypic culture experiments. Antibodies recognizing Pericentrin (ab4448, Abcam), Lamin A/C (2032, Cell Signaling) and V5 (V8137, Sigma; R960-25, Life Technologies) were used. Hoechst 33342, phalloidin, and secondary antibodies labeled with Alexa Fluor 488 nm, 546 nm, 647 nm or 680 nm (Invitrogen) and IR Dye 800CW (Li-Cor Biosciences) were used.

### Transfection of siRNAs

Cells were transfected with 50 nM of siRNA using RNAiMax transfection reagent (Invitrogen) for 24-48 h. The siRNAs were from Dharmacon and Sigma. Cells in all conditions designated as “Control” were transfected with a pool of siRNAs that does not target human genes. Pools of at least 3 siRNAs were used unless otherwise indicated. The details of the sequences for each siRNA used are in the Supplemental Data (Supplementary Table S3).

### Quantitative real-time PCR

A list of primers used is included in the Supplemental Data (Supplementary Table S4). Total RNA was isolated with a GenElute Mammalian Total RNA Miniprep Kit (Sigma,) and converted to cDNA using the iScript cDNA Synthesis Kit (Bio-Rad). Applied Biosystems TaqMan Gene Expression Assays were performed with 20 ng of cDNA using an Applied Biosystems 7500 Real-Time PCR System. GAPDH and specific transcript levels for each transfection condition were measured in triplicate. The ΔΔCT method was applied to quantify relative gene expression [57].

### Quantification of LCPs

Cells were transfected with siRNAs for 24 h and then re-plated onto an ECM consisting of 5 mg/ml Matrigel and 2.1 mg/ml Collagen I. Cells were overlaid with growth media containing 2% Matrigel. Twenty-four hours after plating onto the ECM, the dimensions of at least 40 cells per field of view were evaluated. At least 2 fields of view were evaluated for each experiment, for at least 80 cells total analyzed in each experiment. Cells with ratio of length over width (L/W) >2 were considered to have formed LCPs. The percentage of cells with LCPs is the number of cells with a L/W ratio >2 divided by the total number cells.

### Vertical invasion of tumor cells into ECM

10,000 cells (consisting of H2B:GFP- and H2B:mCherry-labeled cells mixed at a 1:4 ratio) were reverse transfected in duplicate in a 96-well plate with 50 nM siRNAs using RNAiMax transfection reagent (Invitrogen). The use of 2 colors allows for a more accurate quantification of the total cell number using image analysis. After 48 h, media was replaced with 50 μl Matrigel/Collagen matrix (5 mg/ml Matrigel and 2.1 mg/ml Collagen I, unless indicated otherwise), followed by growth media, and incubated at 37°C. Forty-eight hours after ECM addition, cells were imaged with a 20X objective on a Perkin Elmer Ultraview spinning disk confocal microscope equipped with a CCD camera (Hamamatsu). Thirty-one z -slices at 5-μm intervals over a total span of 150 μm were acquired for 10 x,y positions per well that were randomly selected by Volocity software (Perkin Elmer). At least 2 wells (20 x,y positions total) were imaged per condition in each experiment. ImageJ software (NIH) was used to process images into a 3-dimensional stack and quantify invasion. Relative invasion was calculated by using the spot identification feature to count the total number of H2B:mCherry-labeled cells that had migrated 30 μm or more above the monolayer in the reconstructed 3-dimensional images. To correct for possible differences in proliferation rates, the total relative cell number for each well was determined by counting the number of H2B:GFP-labeled cells in the monolayer. The number of invading cells was then divided by the number of monolayer cells to determine the normalized invasion for each well. To determine the relative invasion for each experiment, the normalized invasion value for each condition was divided by the mean normalized invasion for the control condition for the experimental replicates.

### Immunoblotting

Cells were lysed in RIPA buffer supplemented with a protease inhibitor cocktail (Calbiochem) as described [58]. Equal amounts of protein were separated by SDS-PAGE, transferred to Immobilon-FL polyvinylidene fluoride (PVDF) transfer membrane (Millipore), and immunostained. Immunoblots were visualized using an Odyssey infrared scanner (LI-COR). Immunoprecipitations were performed as described [59].

### Immunofluorescence

Cells were fixed and immunostained as described [58]. Images were acquired on a Zeiss LSM700 confocal microscopes in TIFF format. Images were arranged using Photoshop CS6 and Illustrator 6 (Adobe). Where indicated, images “masks” were created with Fiji software using maximum intensity projections of each image. For each projection, actin signals were thresholded for brightness and then converted to binary masks. Immunohistochemical staining of lungs to visualize SUM159T metastases was performed as described [7]. Briefly, formalin-fixed, paraffin-embedded tissue sections were deparaffinized in xylene and rehydrated in a series of alcohol washes. Antigen retrieval was performed by boiling slides in sodium citrate pH 6.0. Slides were blocked in 20% Aquablock (Abcam, Cambridge, MA) in TBS before being incubated overnight with the indicated antibodies, followed by species-specific secondary antibodies conjugated to AlexaFluor488, AlexaFluor546, or AlexaFluor647, and counterstained with Hoechst.

### Time-lapse imaging

Imaging was performed using a Zeiss LSM700 laser scanning confocal microscope enclosed in a 37°C chamber supplemented with humidified CO_2_ (Solent). Images were acquired with a 20x objective (Zeiss) using ZenBlack software (Zeiss) and analyzed with Image J. At least 8 different x,y coordinates were imaged in parallel. Heat maps were generated with ImageJ software. For each image, the green (LifeAct) channel was thresholded for 7 different brightness values at each time point. The first threshold (shown in purple) corresponds to the lowest pixel intensity, while subsequent thresholds correspond to increasingly higher pixel intensities occurring at equally spaced intervals.

### Lentivirus production and infection

For stable RNAi-mediated knockdown of CTAs, SUM159 trailblazer cells, were infected with shRNA clones in the MISSION pLKO.1-puro vector or control vector (SHC001) purchased from Sigma-Aldrich (St. Louis, MO). The shRNA sequences are detailed in Supplementary Table S3. Lentiviral particles were generated by co-transfecting HEK293 cells with shRNA constructs, plus pMDL-, RSV-REV-, and VSVG-expressing packaging plasmids. Transfections were carried out with Lipofectamine 2000 transfection reagent, in accordance with the manufacturer's protocols (Invitrogen, Carlsbad, CA). SUM159T cells expressing GFP were infected with the CTA targeting shRNA or control lentiviruses and then reinfected after 24 hours. Stable cell lines were selected and maintained with 5μg/ml puromycin.

### Xenograft experiments

Age-matched female NOD/SCID mice were used for all in vivo experiments. When possible, littermates were housed together. NOD/SCID mice were obtained from The Jackson Laboratory (Bar Harbor, ME), and bred and maintained under specific pathogen-free conditions in a barrier facility (UTSW, Dallas, TX). All experiments were approved by the Institutional Animal Care and Use Committee performed in compliance with the relevant laws and institutional guidelines of the University of Texas Southwestern Medical Center, Dallas, TX. For spontaneous metastasis experiments, 500,000 SUM159-trailblazer cells stably expressing GFP, and either shSPANX-A/C/D, shCTAG2 or control pLKO.1 constructs were injected into the #4 fat pad of 6-8-week old female NOD/SCID mice. Tumor volumes were measured using calipers. After 42 days, both tumors and lungs were excised, and lung metastases were quantified ex vivo with a fluorescent dissecting microscope by counting the number of clusters of at least 1 GFP expressing cell for micrometastases and 50 GFP expressing cells for macrometastases in at least 3 fields of view per lung. The number of metastases for each set of lungs was then normalized to the corresponding primary tumor weight.

### Breast cancer patient survival analysis

The correlation between SPANX-A/C/D expression and breast cancer patient survival time was performed using the KM-plotter meta-analysis database [60]. patients were stratified into “SPANX-A/C/D-low” and “SPANX-A/C/D-high” groups based on the upper quartile of SPANX-A/C/D expression (Probe ID 220922). ER status was judged by mRNA expression. Survival differences were compared by log-rank test.

### Mass spectrometry

Proteins from SPANX-C immunoprecipitates and proteins that interacted with protein-A sepharose beads were run approximately 10 mm into SDS/PAGE gels. The samples were visualized with Coomassie Blue, removed with a fresh razor blade and transferred to a microcentrifuge tube. The samples were then digested with trypsin overnight and analyzed by reverse phase LC-MS/MS.

### Analysis of gene expression

Significance Analysis of Microarray (SAM) [61] was a new analysis performed on mRNA expression data available at the GEO (GSE58643); [7]. The mRNA expression data (Human HT-12 v4 Expression BeadChip, Illumina Inc.) for cell lines was processed with a model-based background correction approach [62], quantile-quantile normalization and log2 transformation. A threshold of ≥ 4-fold increase in expression in SUM159T cells compared to SUM159O cells with a FDR of <5% was used. Median values of replicate probe sets for the same genes were used to summarize expression values for each gene.

### Statistical methods

Data was analyzed by two-tailed Student's t-test (Graphpad Prism) with the exception of patient survival differences, which were analyzed by log-rank test. P-values < 0.05 were considered significant.

## SUPPLEMENTARY FIGURES, TABLES AND VIDEO












