# Supplementary material for: The cancer-testis antigens SPANX-A/C/D and CTAG2 promote breast cancer invasion
Source: Oncotarget. 2016 Feb 15;7(12):14708–26. doi: 10.18632/oncotarget.7408 (PMC4924746; doi:10.18632/oncotarget.7408)
Supplement: Supplementary file 1 [file oncotarget-07-14708-s001.pdf]

SUPPLEMENTARY FIGURES, TABLES AND VIDEO

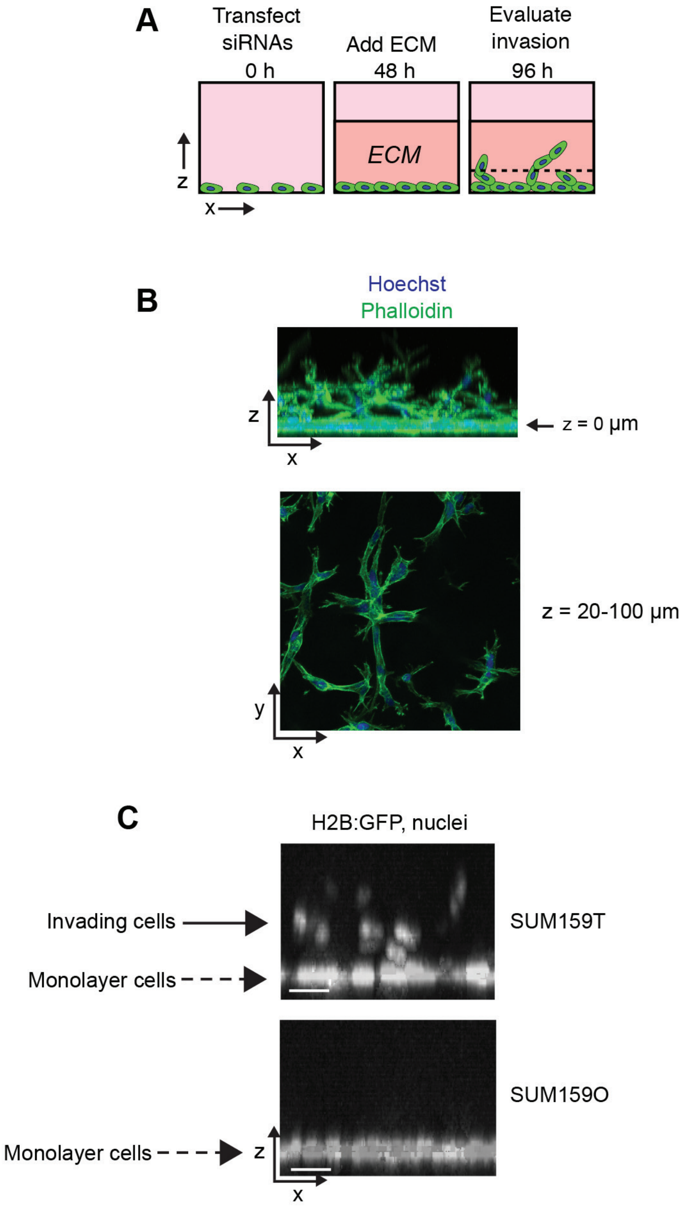

Supplementary Figure S1: A. Graphic showing the "vertical invasion" assay used to determine the relative invasiveness of tumor cells into the ECM. **B.** Images show SUM159T cells invading into the ECM in the vertical invasion assay. The top image shows an x-z view after individual confocal slices are assembled into a 3-dimensional image stack. The bottom image shows an x-y view from between 20-100 µm of the same image stack. This view shows that the SUM159T cells collectively invade as cohesive groups of cells. **C.** Images show x-z projections of SUM159T and SUM159O cells 48 h after the addition of ECM. The fluorescent signal H2B:GFP (nuclei) is shown. The SUM159T cells show enhanced invasion into the ECM relative to the sibling SUM159O cells. Scale bar, 30 µm. See ref. 7 for a detailed analysis of the invasive traits of the SUM159T and SUM159O cells.

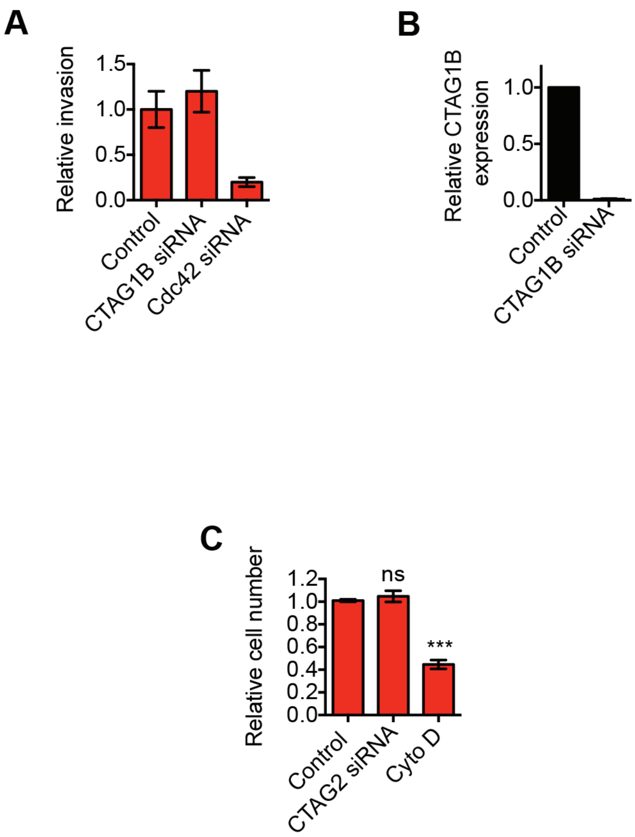

Supplementary Figure S2: A. Graph shows the relative invasion of transfected SUM159T cells ≥ 30 μm into the ECM. The number of invasive cells is normalized to the total cell number in the field of view for each condition, which controls for any variations in cell number. Relative invasion equals the normalized invasive value of the cells transfected with the CTAG1B targeting siRNA pool divided by the mean normalized invasion from the independent experiments for the "control" cells transfected with a pool of 4 siRNAs that do not target human genes. Mean ± range of 2 independent experiments is shown. **B.** Graphs show relative expression of CTAG1B as determined by q-PCR in SUM159T cells transfected as indicated (mean ± range, n=2). **C.** Graph shows the relative number of SUM159T cells transfected with control CTAG2 siRNA pools for 96 h. Cyto D= SUM159T cells grown in parallel and treated with 2 μM starting 48 h after plating and imaged 48 h later (96 h total). Cell number was determined by counting the number of nuclei as described for the vertical invasion assay. Relative cell number equals the the cells transfected with the CTAG1B targeting siRNA pool or treated with cytochalasin D divided by the mean normalized cell number for"control" cells transfected with a pool of 4 siRNAs that do not target human genes. Mean ± standard deviation (SD) of at least 5 biological replicates from 3 independent experiments is shown. ***p< 0.001, ns= not significant, unpaired Student's t test.

Supplementary Table S1: List of genes that have ≥ 4-fold increased expression with a FDR <5% in invasive SUM159T cells relative to non-invasive SUM159O cells.

See Supplementary File 1

Supplementary Table S2: List of proteins identified by LC-MS/MS that immunoprecipitated with SPANX-C:V5.
Proteins that were ≥2-fold enriched in the SPANX-C immunoprecipitates compared to control immunoprecipitates in both replicates and had spectral counts ≥ 10 shown.

See Supplementary File 2

Supplementary Table S3: Sequences of siRNAs used.

See Supplementary File 3

Supplementary Table S4: List of primers used for q-PCR.

See Supplementary File 4

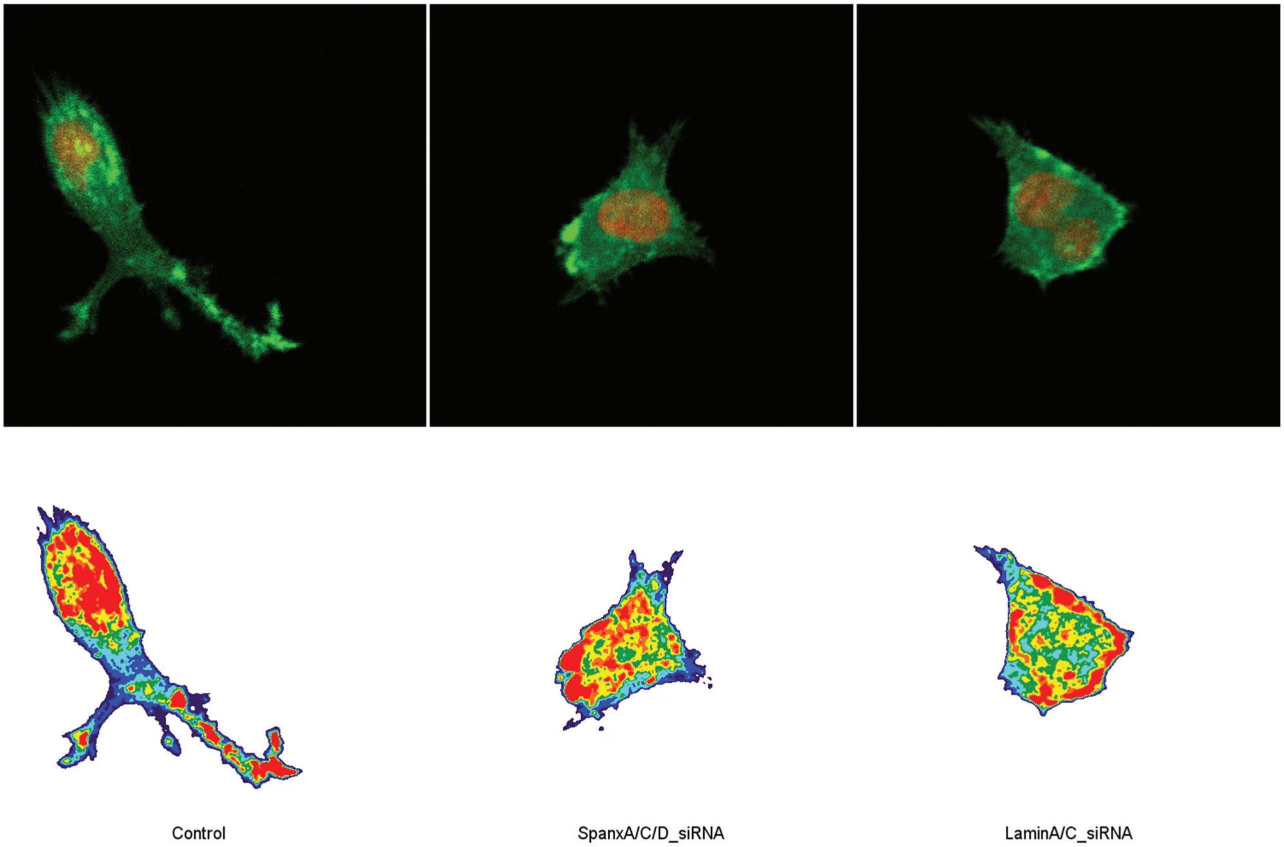

Supplementary Video S1: Time-lapse imaging of SUM159T-LifeACT:GFP cells transfected with SPANX-A/C/D or Lamin A/C siRNAs. Fluorescent images were acquired at 3 min intervals over a span of 1 h. LifeACT:GFP is shown in the upper panels. The bottom panels show the LifeACT:GFP signal converted into a heat map to better visualize differences in signal intensity. This video corresponds with montage of time-lapse images of LifeACT-GFP shown in Figure 3B.
